# Neutrophil dynamics in surgical wounds – A novel role of interleukin-7^☆^

**DOI:** 10.1016/j.ynpai.2025.100199

**Published:** 2025-09-21

**Authors:** Annika Biesold, Philipp Burkard, Ankita Rawat, Laura Cyran, Patrick Meybohm, Heike Rittner, Michael Briese, Nana-Maria Wagner

**Affiliations:** a University Hospital Würzburg, Department of Anaesthesiology, Intensive Care, Emergency and Pain Medicine, Würzburg, Germany; b University Hospital Würzburg, Institute of Clinical Neurobiology, Würzburg, Germany

**Keywords:** Neutrophils, IL-7, IL-7 Receptor, Chronic pain

## Abstract

Postoperative pain and consequently chronic postsurgical pain remain a significant burden among patients. While neutrophilic granulocytes (neutrophils) are known to play a critical role in wound healing, their precise contribution to pain modulation is not fully understood. The neurotrophin nerve growth factor (NGF), released by fibroblasts and immune cells following injury, is an early mediator of pain development. A companion paper demonstrated that NGF administration to axons of cultured sensory neurons in microfluidic chambers induces upregulation of *Il7*, encoding interleukin-7 (IL-7). However, its role in neutrophil dynamics within surgical wounds remains broadly unclear. In a murine model of laparotomy, we observed that IL-7 was expressed at wound sites and co-localized with sensory nerve endings and infiltrating neutrophils within 24 h post-surgery. Laparotomy triggered neutrophil mobilization from the bone marrow, leading to an influx of both mature and immature neutrophils into the circulation. Flow cytometric analysis further revealed IL-7 receptor (IL-7R) surface expression on neutrophils, with IL-7 stimulation also enhancing the expression of activation and maturation markers on neutrophils in vitro. These findings suggest that NGF-induced IL-7 release from peripheral nerve terminals represents a novel neuroinflammatory mechanism modulating neutrophil dynamics in the surgical wound, potentially impairing wound healing and exacerbating pain. Therefore, targeting IL-7 signalling in peripheral nerves may offer a promising strategy for improving postoperative pain management.

## Highlights

• IL-7 is abundant in laparotomy wounds, proximal to neutrophils and nerve terminals.
• Neutrophils express the IL-7 receptor (IL-7R)
• IL-7 exposure leads to neutrophil activation and maturation in vitro.
• IL-7 as new therapeutic target to improve postoperative pain management.

## Introduction

Chronic pain affects millions of people every year, resulting not only in major restrictions and burdens for the patients but also in considerable costs for society (Dueñas et al., 2016, Stubhaug et al., 2024, Widder et al., 2024). Chronic pain has a variety of origins and can occur after surgery, known as chronic post-surgical pain (CPSP). The genesis of CPSP is multifactorial and depends not only on the type of surgery but also on aspects like nerve damage, acute postoperative pain, or inflammation (Correll, 2017, Schug et al., 2016). Surgical incisions typically cause tissue and nerve damage, followed by activation of immune cells and nociceptive neurons (Echeverria-Villalobos et al., 2023, Schug et al., 2016) and secretion of nerve growth factor (NGF) (Schumacher, 2010). Although not fully understood yet, NGF is suggested to contribute to chronic pain via sensitization and neuronal sprouting (Schmelz et al., 2019). A companion paper published in this special issue observed that the stimulation of axons with NGF results in transcriptional changes with *Il7* mRNA encoding for the interleukin-7 (IL-7) being one of the most significant upregulated transcripts (Koch et al., 2025). IL-7 is a key cytokine in the immune system, essential for the development, survival, and maintenance of T cells and B cells, especially in early life and during immune responses. Recombinant IL-7 is under investigation for immune reconstitution in cancer, HIV or sepsis. Apart from the function on the adaptive immune system it has long been known to influence neutrophils. It is assumed that neutrophils like other immune cells express the IL-7 receptor (IL-7R) (Chen et al., 2021). However, the role of this receptor in neutrophil biology has attracted less attention. Initial evidence suggests that IL-7 exerts an influence on neutrophils. Several studies have reported that injecting IL-7 into mice induces neutrophilia (Damia et al., 1992, Grzegorzewski et al., 1994), and blood smears following the administration of human recombinant IL-7 showed an increase in murine immature neutrophils (Faltynek et al., 1992). Moreover, it was reported that IL-7 influences the attraction of neutrophils (Kasten et al., 2010). Neutrophils are among the first immune cells recruited to the wound site, playing a crucial, yet incompletely understood role in wound healing and pain modulation. Current evidence suggests that neutrophils play a dual role in wound healing and chronic pain development (Wilgus et al., 2013, Huerta et al., 2024). They contribute to host defence by eliminating pathogens, orchestrating the recruitment of additional immune cells, and releasing pro-angiogenic and growth factors that promote tissue repair. However, their antimicrobial peptides and enzymes exhibit limited specificity, which can lead to collateral damage to host tissues. Moreover, their secretion of prostaglandin E2 (PGE2) and interleukin-18 (IL-18) can stimulate nociceptive neurons and contribute to sensitization. Dysregulation in neutrophil recruitment, apoptosis, or clearance can exacerbate tissue injury, perpetuate local inflammation, influence pain perception and potentially contribute to the transition from acute to chronic pain (Wilgus et al., 2013, Huerta et al., 2024). This study aimed to analyse the dynamics of IL-7 secretion and neutrophil recruitment following surgery and to investigate the effects of IL-7 on neutrophil activation, maturation, and IL-7R expression.

## Methods and materials

### Animals

C57BL/6J wild-type mice (Charles River, Sulzfeld, Germany) were maintained under pathogen-free conditions. All procedures complied with the German Animal Welfare Act and were approved by local authorities.

### Median laparotomy

Mice were anesthetized via intraperitoneal (i. p.) injection of ketamine (100 mg/kg b. w.) and xylazine (10 mg/kg b. w.). Following aseptic preparation, a 1 cm median abdominal incision was performed, and the peritoneum and skin were sutured using PROLENE^TM^, with additional fixation via 3 M Vetbond^TM^. Surgery was conducted on a heating plate (37 °C), and postoperative analgesia (butorphanol, 2 mg/kg b. w.) was administered subcutaneously s. c. every 4 h for 12 h.

### Murine organ harvesting

Blood was collected under deep anaesthesia via atrial puncture into EDTA tubes. Spleens were processed through a 70 µm strainer with FACS buffer (1% FCS in PBS), followed by red blood cell lysis (Roche), washing steps and resuspension in FACS buffer. Bone marrow was extracted from femurs and tibias via centrifugation and treated with the lysis buffer before resuspension in FACS buffer. Cell counts were determined using Countess 3 (Invitrogen).

### Skin wound sampling and immunofluorescence staining after laparotomy

Laparotomy skin samples were collected at 24 h alongside control samples from untreated mice. Tissues were snap-frozen in Tissue-Tek® O.C.T. Compound and sectioned at 10 µm thickness. Sections were fixed with 4% PFA, permeabilized and blocked with 10% donkey sera in PBS, 0.3% Triton-X and 0.1% Tween-20 before primary antibody incubation (4 °C, 24 h). After washing steps and secondary antibody staining (RT, 1 h), DAPI (0.5 µg/mL) was applied, and sections were mounted with FluorSave^TM^. For immunostaining, primary antibodies included unlabelled anti-Nefh (Merck Millipore) and anti-IL-7 (PeproTech). Corresponding secondary antibodies were Alexa Fluor 488-conjugated for Nefh and Cyanine 5-conjugated for IL-7 (Jackson ImmunoResearch). Additionally, PE-conjugated anti-IL-7R (CD127) and Alexa Fluor 647-conjugated anti-Ly6G (BioLegend) were used for staining.

### Isolation and stimulation of human neutrophils

Human neutrophils were isolated using the MACSxpress® Whole Blood Neutrophil Isolation Kit and MACSxpress® Separator according to manufacturers’ instructions. For stimulation, agonists (100 ng/mL or 1 µg/mL IL-7, 1 µM fMLP, 100 ng/mL LPS) were added to 500,000 neutrophils in RPMI and incubated for 30 min at 37 °C in the dark Cells were washed with FACS buffer before being stained with fluorescently labelled antibodies for subsequent flow cytometry analysis.

### Antibody cell staining for flow cytometry analysis

For extracellular staining, 500,000 cells per sample were resuspended in an antibody master mix and incubated (30 min, 4 °C). After washing, cells were resuspended in 500 µl of FACS buffer and analysed using the Thermo Fisher Attune NxT. For intracellular staining, the eBioscience™ Foxp3/Transcription Factor Staining Buffer Set (Invitrogen) was used according to manufacturer’s instructions.

Murine cells were stained with Brilliant Violet 421-labelled anti-CD45, Brilliant Violet 510-labelled anti-CD11b, Alexa Fluor 647-labelled anti-Ly6G, PE-labelled anti-CD101, and Brilliant Violet 650-labelled cKit (CD117). All from BioLegend. Antibodies were used in a 1:200 dilution.

IL-7R expression in murine neutrophils was analysed using PE-labelled anti-IL7-R (mouse).

Isolated human neutrophils were stained with FITC-labelled anti-CD3/CD19/CD20/CD56 (dump channel), Alexa Fluor 488-labelled anti-CD115, PE/Cyanine7-labelled anti-CD11b, PacificBlue-labelled anti-CXCR2, Alexa Fluor 700-labelled anti-CD14, PerCP/Cyanine5.5-labelled anti-CD62L, Brilliant Violet 510-labelled anti-CD16, PE-labelled anti-CD97, Alexa Fluor 647-labelled anti-CD66b, and Brilliant Violet 650-labelled anti-CD10. All from BioLegend. Antibodies were used in a 1:200 dilution.

Flow cytometry analysis was performed with FlowJo (ImageJ 2.14.0). Single cells were gated by excluding doublets and viable cells were identified as viability dye negative. Murine neutrophils were identified as CD45+/Ly6G+/CD11b+ and isolated human neutrophils as Dump negative (CD3-/CD19-/CD20-/CD56-/CD115-). The IL-7R expression was analysed via an anti-IL-7Rα antibody and a fluorescence minus one (FMO) control. The maturation and activation state of human neutrophils was assessed via staining for CD10, CD16, and CXCR2 as maturity markers, and CD97, CD66b, CD11b, CD62L, and CD14 as activation markers. For mouse neutrophils Data visualization and statistical analysis were performed using GraphPad Prism (10.1.2).

### Imaging and evaluation of immunofluorescence picture

Images were acquired using an OLYMPUS FV1000 confocal laser scanning microscope (60 × oil, NA: 1.35) with excitation at 405, 473, 559, and 633 nm. Z-stacks were captured at 1 µm step sizes, covering the full tissue depth, with consistent imaging parameters. Additional imaging was performed using a Zeiss Axio Imager.M2 microscope with ApoTome.2 structured illumination (40 × oil, Plan-APOCHROMAT Zeiss), using 405, 488, 555, and 647 nm excitation.

Percentage area analysis was performed using ImageJ (2.14.0) to quantify IL-7, IL-7R, and neutrophil signals in Z-stacks. For each wound section, six randomly selected Z-stacks were analyzed. The analysis involved channel splitting, selecting the target channel, generating a maximum intensity projection (Z-Project), applying a median filter (1 pixel), and using the MaxEntropy automated threshold to quantify signal area coverage.

### Statistics

Statistical analyses were performed using GraphPad Prism (10.1.2). An unpaired *t*-test was used for two-group comparisons. For multiple comparisons of parametric data, a one-way ANOVA with Dunnett’s test or Brown-Forsythe/Welch ANOVA was used. Group means were compared to the control, with significance set at α = 0.05 (* *P ≤ 0.05*, ** *P < 0.01*, *** *P < 0.001*, *** *P < 0.0001*). Data are shown as mean ± standard error of the mean (SEM).

## Results

### IL-7 is found in wounds after laparotomy

**Fig. 1:**
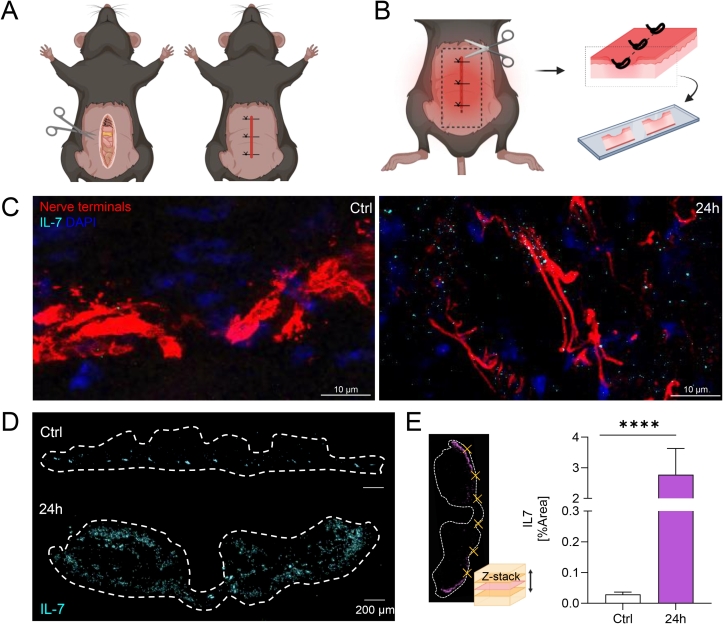
IL-7 is found in wounds after laparotomy. A, B. Schematic illustration of the pre-clinical model of laparotomy in mice (left) and sampling of skin wounds after laparotomy (right). Illustrated with BioRender. C. Immunofluorescence staining of skin cryosections from control (Ctrl) skin and laparotomy wound taken 24 h after surgery showed neurons (stained with anti-Nefh, red) surrounded by IL-7 (cyan). Nuclei were stained with DAPI (blue). Z-stacks were acquired using an OLYMPUS FV100 confocal microscope with a 60x objective. Scale bar 10 µm. D. Immunofluorescence overview images of whole wound sections (see illustration B.) stained for IL-7 (cyan). Wound sample was taken 24 h after laparotomy and is compared to control skin. Overview pictures were imaged via Zeiss Axio Imager.M2 equipped with ApoTome2 and a 40x objective. Scale bar 200 µm. E. Area coverage [%] of IL-7 within immunostained cryosections of whole wound skin sections. For each wound section, six randomly selected FOVs (Z-stacks) were analyzed. Bars represent mean ± SEM. Nonparametric one-way ANOVA with Dunnett́s multiple comparison test. **** P < *0.0001.* (For interpretation of the references to colour in this figure legend, the reader is referred to the web version of this article.)

NGF administration to axons of sensory neurons in vitro results in the local production and release of IL-7 (Koch et al., 2025). In vivo, NGF is released by fibroblasts, keratinocytes and immune cells following injury (Schumacher, 2010), suggesting its presence at wound sites where it potentially leads to IL-7 secretion as well. To test this, we performed laparotomy in wild-type mice as a preclinical model (Fig. 1A, B). Skin wound samples were taken and stained for IL-7 by fluorescently labelled antibodies. Confocal Z-stack imaging of immunofluorescence-stained skin wound cryosections revealed the presence of IL-7 within the laparotomy wound. IL-7 (cyan) was detected in close proximity to peripheral neurons (red) (Fig. 1C) 24 h after surgery. Moreover, tile scans, covering the complete wound, showed a strong visual increase in IL-7 immunoreactivity in laparotomy samples compared to non-operated control samples (Fig. D). When quantifying the area coverage of the IL-7 signal in 6 randomly selected field of views (FOVs) the percentage of covered area was significantly higher in laparotomy wounds after 24 h compared to skin samples from untreated mice (Fig. 1D, E).

### IL-7 in wounds co-localizes with accumulated neutrophils after laparotomy

**Fig. 2:**
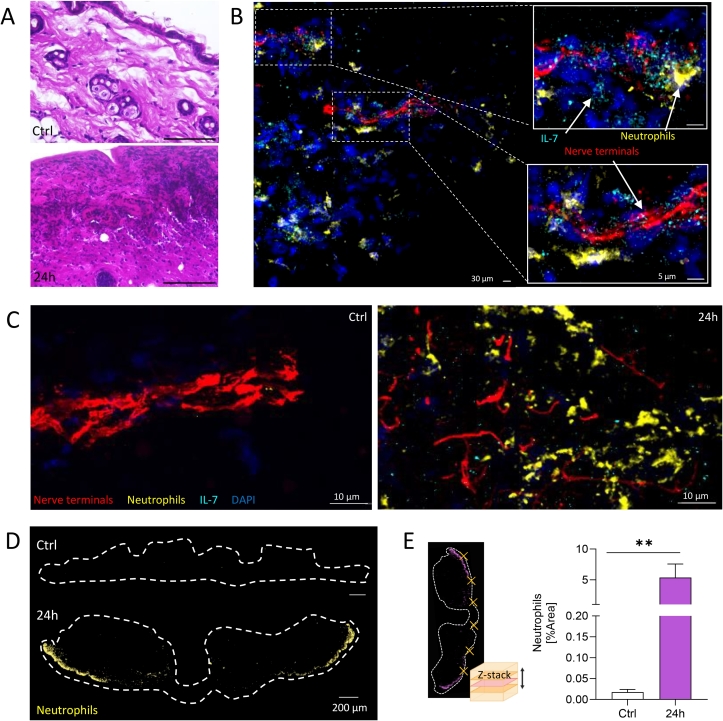
IL-7 in wounds co-localizes with accumulated neutrophils. A. H&E stained histological skin sections from laparotomy wounds 24 h post-surgery showing massive cellular infiltrates compared to untreated (control) mice. Scale bar 100 µm. B. Immunofluorescence staining of skin cryosections from laparotomy wounds 24 h post-laparotomy showing close proximity of neurons (stained with anti-NEFH, red), IL-7 (cyran) and neutrophils (stained with anti-Ly6G, yellow). Nuclei were stained with DAPI (blue). Scale bar 30 µm (left) and 5 µm for magnified excerpts. Z-stacks were taken via OLYMPUS FV1000 confocal microscope with a 60x objective. C. Control (Ctrl.) skin and laparotomy wound cryosections at 24 h immunostained for nerve terminals (red), IL-7 (cyran), neutrophils (yellow) and cell nuclei (blue). Z-stacks were taken via OLYPMUS FV1000 confocal microscope 60x objective. Scale bar of 10 µm. (D) Immunofluorescence overview images of whole wound sections stained for neutrophils (yellow). Wound sample was taken 24 h after laparotomy and is compared to control skin. Overview pictures were taken via Zeiss Axio Imager.M2 equipped with ApoTome2 and a 40x objective. Scale bar 200 µm. (E) Percentage area analysis for the presence of neutrophils within immunostained cryosections of whole wound skin sections. For each wound section, six randomly selected FOVs (Z-stacks) were analyzed. Bars represent mean ± SEM. Nonparametric one-way ANOVA with Dunnett́s multiple comparison test. ** P < 0.01. (For interpretation of the references to colour in this figure legend, the reader is referred to the web version of this article.)

Given the potential expression of IL-7R on neutrophils and the established role of IL-7 in promoting neutrophil recruitment, we further examined laparotomy wound sections using H&E and immunofluorescence staining. H&E staining revealed a substantial increase in cellular infiltration within the wound following surgery compared to untreated skin samples (Fig. 2A). Immunofluorescence staining demonstrated the close proximity of neutrophils (yellow), neuronal axons (red), and secreted IL-7 (cyan) (Fig. 2B). Moreover, a marked increase in both, neutrophils and IL-7, was observed 24 h post-surgery compared to control samples (Fig. 2C). Neutrophil infiltration into the wound was further visualized through whole-wound imaging, which showed an increase in neutrophil signal after laparotomy compared to untreated controls (Fig. 2D). Similar to IL-7, quantification of the neutrophil area coverage confirmed the marked neutrophil accumulation at the wound site 24 h post-surgery (Fig. 2E).

### Laparotomy is associated with neutrophil recruitment from the bone marrow

**Fig. 3:**
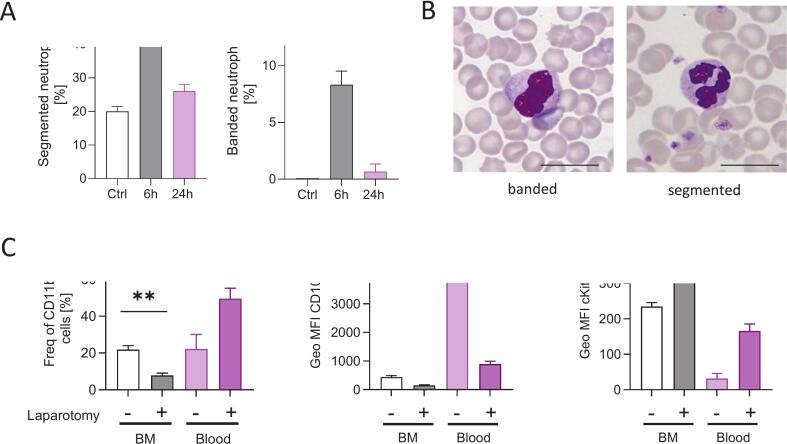
Laparotomy triggers neutrophil recruitment from bone marrow. A. Analysis of blood smears from control and laparotomy mice 6 h and 24 h after surgery showed changes in the percentage of mature (segmented) and immature (banded) neutrophils. Bars represent mean ± SEM. Parametric one-way ANOVA with Dunnett́s multiple comparison test. B. Images of neutrophils with banded (6 h after laparotomy) and segmented nuclei. Scale bar 12 µm. C. Flow cytometric analysis of stained bone marrow (BM) and blood cells from mice before and 6 h after laparotomy showing frequency of CD11b + cells regarding all leucocytes [%] and the geometric mean fluorescence intensity (Geo MFI) of CD101 and cKit (CD117) on CD45 + Ly6G + CD11b + neutrophils. Bars represent mean ± SEM. An unpaired *t*-test was used to compare both treatment groups (control vs. laparotomy) for each analysed compartment (BM and Blood). * P ≤ 0.05, ** P < 0.01.

Blood smears demonstrated a significant mobilization of neutrophils from the bone marrow into the bloodstream following laparotomy compared to control mice. Both mature (segmented) and immature (banded) neutrophils were recruited, with a peak 6 h post-surgery, with mature neutrophils being the majority (Fig. 3A, B). Flow cytometry analysis confirmed the results obtained by the blood smears. Laparotomy induced a reduction in neutrophils within the bone marrow, which translated into an increase of CD11b+ neutrophils in peripheral blood (Fig. 3C, left graph, Suppl. Fig. 1). Furthermore, the overall maturation status of the neutrophil population shifted towards immature, reflected by a significant decrease in the maturation marker CD101 and an increase in the immaturity marker CD117 (cKit) (Fig. 3C, middle and right graph).

### Neutrophils express the IL-7 receptor

**Fig. 4:**
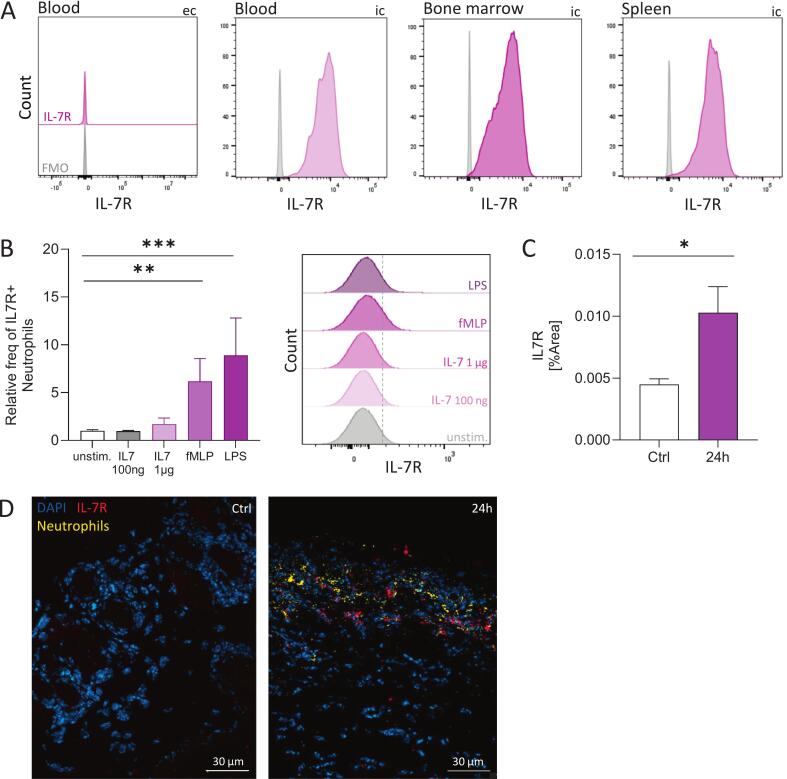
Neutrophils express the IL-7 receptor. A. Histograms of flow cytometry analysis of murine blood, bone marrow and spleen cells gated on neutrophils expressing the IL-7 receptor (IL-7R). Neutrophils were stained extracellulary (ec) and intracellulary (ic) for the IL-7R (pink) and were compared with the respective fluorescence minus one (FMO) control (gray). B. Neutrophils isolated from human blood were stained for the extracellular expression of IL-7R with and without stimulation with different agonists for 30 min at 37 °C. For stimulation IL-7 (100 ng/mL, 1 µg/mL), fMLP (1 µM) and LPS (100 ng/mL) were used. The relative frequency of IL-7R+ neutrophils among all neutrophils is shown. Bars represent mean ± SEM. Parametric one-way ANOVA with Dunnett́s multiple comparison test. ** P < 0.01, *** P < 0.001. The histogram additionally illustrates the altered extracellular expression of the IL-7R on neutrophils after stimulation. C. Area coverage [%] of the IL-7R within immunostained cryosections. For each wound section, six randomly selected FOVs (Z-stacks) were analyzed. Bars represent mean ± SEM. Nonparametric one-way ANOVA with Dunnett́s multiple comparison test. * P < 0.01. D. Immunostaining of wound sections stained for IL-7R (red), neutrophils (Ly6G, yellow) and cell nuclei (DAPI, blue). Wound samples taken 24 h after laparotomy were compared to control skin. Z-stacks were acquired with a Zeiss Axio Imager.M2 equipped with ApoTome2 and a 40x objective. Scale bar 30 µm. (For interpretation of the references to colour in this figure legend, the reader is referred to the web version of this article.)

The expression of the IL-7 receptor (IL-7R) on neutrophils remains unexplored and incompletely understood. To further clarify this aspect, we analysed both membrane bound, extracellular and intracellular IL-7R expression in murine and human neutrophils by flow cytometry and immunofluorescence staining of wound sections. In mice, neutrophils isolated from blood, spleen, and bone marrow predominantly exhibited intracellular IL-7R expression, while extracellular expression was low (Fig. 4A). Notably, extracellular IL-7R expression was inducible upon stimulation. In human neutrophils, stimulation with various agonists for 30 min at 37 °C increased extracellular IL-7R levels (Fig. 4B). Furthermore, area coverage analysis of IL-7R signal in wound sections 24 h post-laparotomy revealed a significant upregulation of extracellular IL-7R compared to control sections, as confirmed by immunostaining (Fig. 4C, D).

## IL-7 exposure leads to phenotypic changes of neutrophils in vitro

**Fig. 5:**
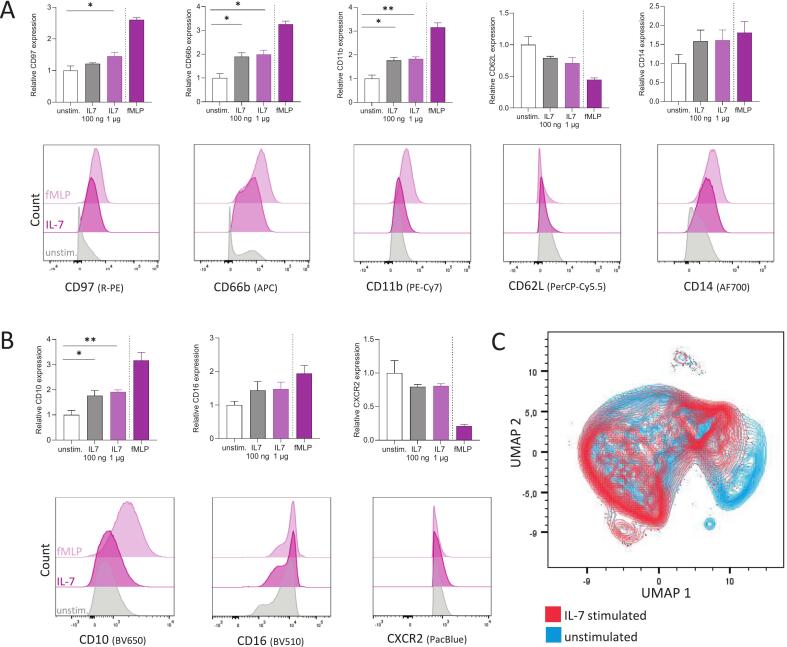
IL-7 exposure leads to phenotypic changes of neutrophils in vitro. A, B. Expression of human neutrophil activation (A) and maturation (B) markers after IL-7 (100 ng/mL, 1 µg/mL) and fMLP (1 µM) stimulation for 30 min at 37 °C compared to unstimulated control. Relative expression was shown as fold change over unstimulated control. The shift of activity markers after IL-7 (1 µg/mL) and fMLP (1 µM) stimulation compared to the unstimulated control were additionally shown in histograms. Isolated human neutrophils were defined as Dump- (CD3-/CD19-/CD20-/CD56-/CD115-). Bars represent mean ± SEM. fMLP served as positive control and was excluded from the statistical analysis. Unpaired two-tailed *t*-test was used. * P ≤ 0.05, ** P < 0.01. C. Multidimensional UMAP illustration giving an overview of all stained neutrophil activation (A) and maturation (B) markers after IL-7 (1 µg/mL) stimulation (red) in comparison to unstimulated neutrophils (blue). (For interpretation of the references to colour in this figure legend, the reader is referred to the web version of this article.)

Finally, we investigated the effects of IL-7 stimulation on the phenotype of isolated human neutrophils, focusing on their maturation and activation state. Neutrophils were stimulated with IL-7 for 30 min at 37 °C and subsequently analysed by flow cytometry. Compared to unstimulated controls, IL-7-stimulated neutrophils increased expression of activation and maturation markers. Precisely, the activation markers CD97, CD66b, and CD11b showed a significant increase following IL-7 stimulation, while – in a trend − CD14 was upregulated and CD62L decreased (Fig. 5A). The maturation markers, CD10 and CD16 were elevated after IL-7 stimulation, whereas CXCR2 demonstrated a trend toward downregulation (Fig. 5B). UMAP analysis further supported these findings by showing the presence of a distinct shift of the population when comparing the expression pattern of unstimulated and IL-7 stimulated neutrophils (Fig. 5 C).

## Discussion

IL-7, primarily recognized for its role in lymphocyte development and homeostasis, has recently emerged as a factor of interest in neutrophil biology (Chen et al., 2021). Our findings show IL-7 close to nerve endings in laparotomic wounds, which we propose to be the source of IL-7 after NGF-stimulation (Koch et al., 2025). To explore the potential effects of IL-7 on neutrophils, we first assessed IL-7 receptor (IL-7R) expression in these cells. Flow cytometric analysis revealed that IL-7R was primarily localized intracellularly in resting neutrophils, with minimal surface expression. However, membrane IL-7R expression was induced upon stimulation with LPS and fMLP. These findings are consistent with previous in vitro studies demonstrating that myeloid cells can alter the expression patterns of intracellular and extracellular interleukin receptors in response to stimulation (Hodge et al., 2000, Wu et al., 2008). In contrast, direct stimulation with IL-7 induced only a trend of increased extracellular IL-7R expression. Thus, in the inflammatory environment of the wound, its expression may be influenced by other pro-inflammatory mediators. Immunohistochemical analysis of cryosections further revealed that IL-7R expression was detectable 24 h after laparotomy. These findings suggest that neutrophils initially infiltrating the wound may express little to no extracellular IL-7R. However, exposure to inflammatory mediators within the wound microenvironment likely stimulates IL-7R upregulation. IL-7, released by peripheral nerve endings, may then influence their function and behavior within the inflammatory milieu, indicating potential immunoregulatory roles beyond its well-characterized effects on lymphocytes.

One proposed effect of IL-7 on neutrophils is its ability to promote their recruitment to sites of injury. Our findings showed close proximity of IL-7 and neutrophils within the laparotomy wound. Our in vitro experiments demonstrated that IL-7 exerts a significant influence on neutrophil activation and maturation. Upon stimulation, neutrophils showed a marked upregulation of the activation markers CD97, CD66b, and CD11b. Additionally, the observed trend of decreased CD62L expression further supports a shift toward an activated state. With respect to maturation, the significant upregulation of CD10 and CD16 indicates that IL-7 promotes not only neutrophil activation but also maturation. Further studies exploring the role of IL-7 in mediating neutrophil effector functions, particularly in pre-stimulated cells under inflammatory conditions, could provide valuable insights into its immunomodulatory potential.

While IL-7R has been identified in various myeloid cells (Park et al., 1990), recent evidence suggests that human neutrophils constitutively express the interleukin receptor gamma chain (γ-chain) of IL-7R. This γ-chain is a shared component of multiple interleukin receptors, including IL-2R, IL-4R, IL-9R, and IL-15R (Girard and Beaulieu, 1997). However, the expression dynamics of the IL-7Rα (CD127) subunit on neutrophils remain poorly understood. Girard et al. reported that IL-7Rα was not detectable. However, their study relied solely on standard extracellular staining methods and did not assess intracellular IL-7Rα expression. Our stainings for IL-7Rα are consistent with their findings, as we observed minimal extracellular IL-7Rα expression on neutrophils. However, intracellular staining revealed substantial levels of IL-7Rα, suggesting that this receptor subunit may be primarily stored within neutrophils and potentially mobilized to the cell surface under specific stimulatory conditions. Interleukin receptor subunits can exhibit distinct expression patterns in intracellular and extracellular compartments, as has been reported for other cytokine receptors (Hodge et al., 2000). These observations highlight the distinct and dynamic expression patterns of interleukin receptor subunits across different cell types, including T- and B-cells. Immature T- and B-cells both express IL-7R, but while naïve T-cells maintain IL-7R expression until T-cell receptor engagement and can later re-express it, naïve B cells lose IL-7R expression entirely upon maturation (Colpitts et al., 2009, Clark et al., 2014, Pongratz et al., 2014). Similar regulatory mechanisms may govern IL-7Rα expression on neutrophils. Moreover, previous studies have shown that the interleukin receptor expression on resting cells is inducible upon stimulation with agonists like LPS (Hodge et al., 2000). This also applies for our data showing that the extracellular IL-7Rα expression on neutrophils is inducible with fMLP and LPS. Furthermore, it is proposed that interleukin receptor chains can be stored intracellularly before being translocated to the cell surface upon activation (Hodge et al., 2000). Our detection of substantial intracellular IL-7Rα expression in neutrophils suggests a similar mechanism of intracellular storage, allowing for rapid mobilization and activation in response to environmental cues. Given that neutrophils are among the first immune cells to arrive at the wound site, this ability to swiftly respond to IL-7 in the inflammatory milieu may be crucial for their function in early immune responses. Further investigations into the regulatory mechanisms governing IL-7Rα translocation and its functional consequences in neutrophil activation will provide deeper insights into its role in post-surgical inflammation and immune modulation.

Blood smears and flow cytometry analyses following laparotomy revealed a mobilization of mature and immature neutrophils from the bone marrow into the bloodstream at 6 h post-surgery. 24 h after surgery we observed a peak of neutrophil recruitment within the wound. Previously, Kim et al. (Kim et al., 2008) reported a correlation between increased systemic neutrophil levels and enhanced wound infiltration supporting our observations. Recent studies have provided evidence that neutrophils play a dual role in wound healing and chronic pain development, potentially hindering the resolution of inflammation and promoting the transition from acute to chronic pain (Wilgus et al., 2013, Huerta et al., 2024). The transition from the inflammatory phase to the proliferative phase is primarily driven by the phagocytosis of apoptotic neutrophils by macrophages. The recruitment of neutrophils to the wound site has been associated with the upregulation of key genes involved in wound healing and stimulating the proliferation of keratinocytes and fibroblasts (Theilgaard-Mönch et al., 2004, Wilgus et al., 2013). However, activated neutrophils additionally release reactive oxygen species (ROS), neutrophil extracellular traps (NETs), and proteolytic enzymes which can cause collateral damage to the surrounding host tissue. Therefore, an excessive or prolonged neutrophil response following injury can have detrimental effects like sustained inflammation and delayed healing (Wilgus et al., 2013). This phenomenon has been linked to the development of chronic wounds in conditions such as vascular diseases and diabetes (Pierce, 2001). Similarly, studies have shown that excessive neutrophil infiltration is associated with impaired healing in both full-thickness skin wounds and volumetric muscle loss injuries in the quadriceps (Lu et al., 2024). In addition, an overabundance of neutrophils leads to the excessive secretion of pro-inflammatory cytokines, which in turn stimulate neutrophils to release additional ROS, NETs, and cytokines. Enhanced clearance of inflammatory cells from injured tissues instead, reduces excessive inflammation and accelerates healing (Wilgus et al., 2013, Wong et al., 2015, Lu et al., 2024). Moreover, IL-7 activates signalling pathways upon binding to the IL-7R, leading to the upregulation of anti-apoptotic and downregulation of pro-apoptotic molecules. This dual regulation favours cell survival by enhancing resistance to apoptosis (Huang et al., 2021). In the context of laparotomy wounds, increased expression of IL-7R on neutrophils may result in a heightened resistance to apoptosis, thereby extending their presence at the wound site. The prolonged persistence of neutrophils could exacerbate tissue damage, as these cells continue to release proteases, ROS, and other molecules that contribute to inflammation and delay the resolution of the wounds and pain. This highlights the importance of a balanced neutrophil dynamic to prevent prolonged tissue damage and promote healing.

Prolonged inflammation not only impedes wound healing but also plays a crucial role in the modulation of pain. Inflammatory pain typically results from heightened sensitivity and lowered activation thresholds of nociceptors due to the presence of inflammatory mediators (Jain et al., 2024). Following tissue injury, inflammatory mediators are released by activated nociceptors as well as non-neuronal cells, including neutrophils that infiltrate the site of injury. Neutrophils release mediators such as prostaglandin E2 (PGE2) and interleukin-18 (IL-18) which may contribute to the maintenance of chronic pain by stimulating nociceptive neurons and promoting inflammatory responses (Huerta et al., 2024). This can result in transcriptional and translational upregulation of pro-nociceptive peptides and ion channels and consequently heightened nociceptor depolarization, initiating both peripheral and central sensitization. Jain et al. (2024) demonstrated that nociceptor sensitization occurred after paw-skin injury in a murine model, with the temporal progression of pain development correlating with the recruitment of neutrophils to the wound site. This observation supports the hypothesis that neutrophils play a significant role in the development of inflammatory pain. During inflammation, neutrophils release pro-inflammatory cytokines, such as tumor necrosis factor-alpha (TNF-α) and IL-1β (Giambelluca et al., 2014, McColl et al., 1992), which further promote the production of pro-algesic agents, including NGF (Rittner et al., 2008). Persistent inflammation, therefore, may also lead to sustained NGF production, which in turn enhances the responsiveness of nociceptors. Chronic exposure to NGF can induce prolonged peripheral and, notably, central sensitization, promoting neuroplastic changes in the nervous system that amplify sensitivity to pain signals and potentially lead to chronic pain conditions. Indeed, elevated NGF levels have been observed in animal models of chronic pain, as well as in humans suffering from chronic pain.

## Conclusion

Our study identifies neuronal IL-7 as a novel modulator of neutrophil behavior in the postoperative wound environment. While an early and robust neutrophil response is essential for promoting tissue repair and preventing the chronification of pain (Jain et al., 2024), our findings support the growing evidence that prolonged or dysregulated neutrophil activity can perpetuate inflammation and promote the transition from acute to chronic pain (Wilgus et al., 2013, Lu et al., 2024, Huerta et al., 2024).

We propose that IL-7, released from peripheral neurons following tissue injury, interferes with the physiological regulation of neutrophil dynamics by enhancing their activation and possibly sustaining their presence at the wound site. This disturbance may contribute to a prolonged inflammatory state that impairs healing and sensitizes nociceptive pathways.

Importantly, the aim should not be the complete abrogation of acute inflammation, which is critical for effective wound healing, but rather to restore neutrophil responses to a physiological balance. By fine-tuning neutrophil dynamics, therapeutic targeting of IL-7 signalling could promote resolution of inflammation, support wound repair, and prevent the development of chronic post-surgical pain. Future studies should explore the translational potential of modulating neuronal IL-7 in clinical settings of surgery-associated inflammation and pain.

## CRediT authorship contribution statement

**Annika Biesold:** Writing – original draft, Visualization, Methodology, Investigation, Formal analysis. **Philipp Burkard:** Writing – review & editing, Supervision, Methodology, Investigation, Conceptualization. **Ankita Rawat:** Writing – review & editing, Methodology, Investigation. **Laura Cyran:** Supervision, Methodology, Investigation. **Patrick Meybohm:** Writing – review & editing, Resources. **Heike Rittner:** Writing – review & editing, Resources, Project administration, Funding acquisition. **Michael Briese:** Writing – review & editing, Resources, Project administration, Funding acquisition. **Nana-Maria Wagner:** Writing – review & editing, Writing – original draft, Supervision, Resources, Project administration, Funding acquisition, Conceptualization.

## Declaration of competing interest

The authors declare that they have no known competing financial interests or personal relationships that could have appeared to influence the work reported in this paper.

## Data Availability

Data will be made available on request.
