# Supplementary figures and images for: Neutrophil dynamics in surgical wounds – A novel role of interleukin-7
Source: Neurobiol Pain. 2025 Sep 21;18:100199. doi: 10.1016/j.ynpai.2025.100199 (PMC12508816; doi:10.1016/j.ynpai.2025.100199)

Figure 1

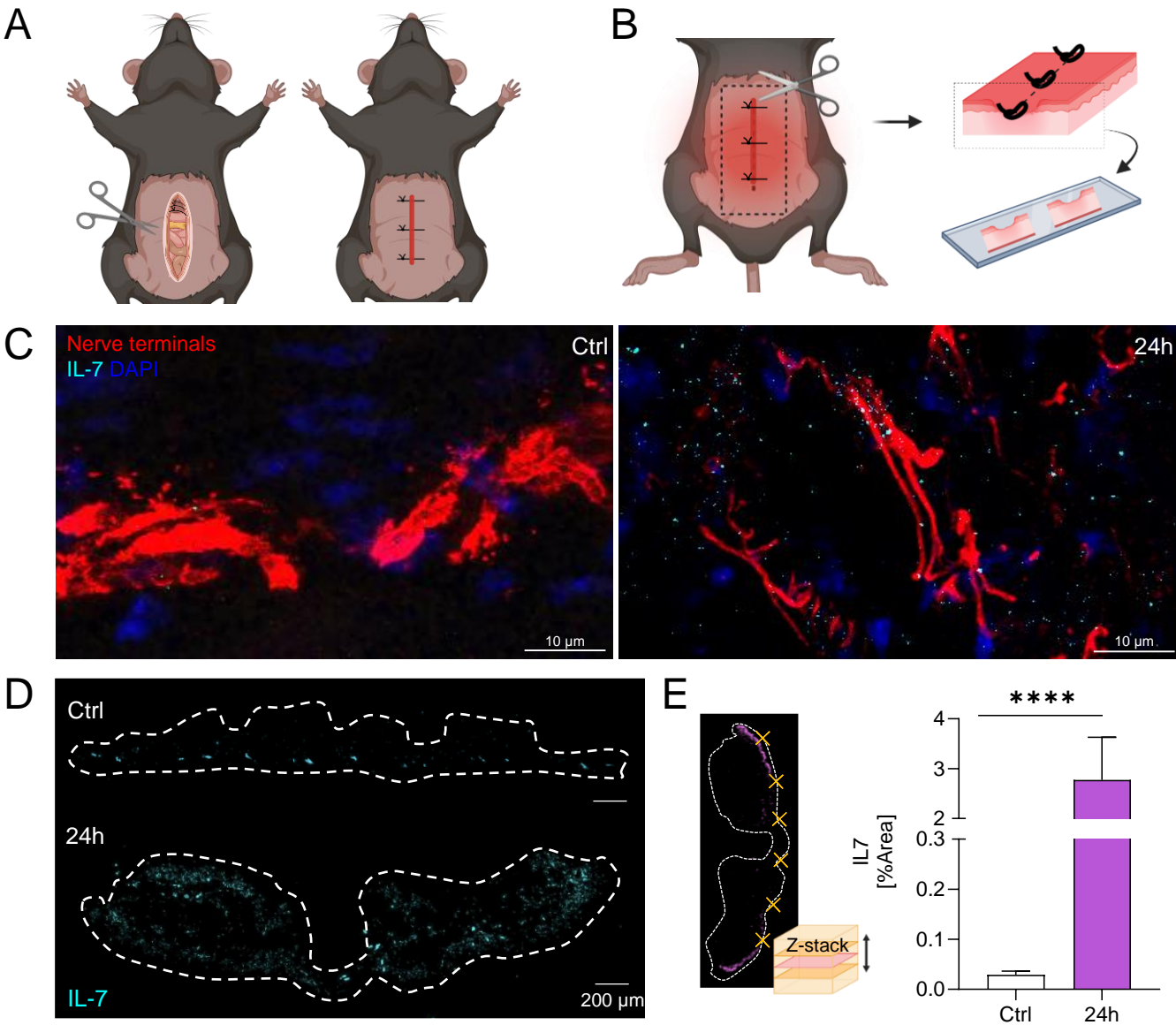

Figure 2

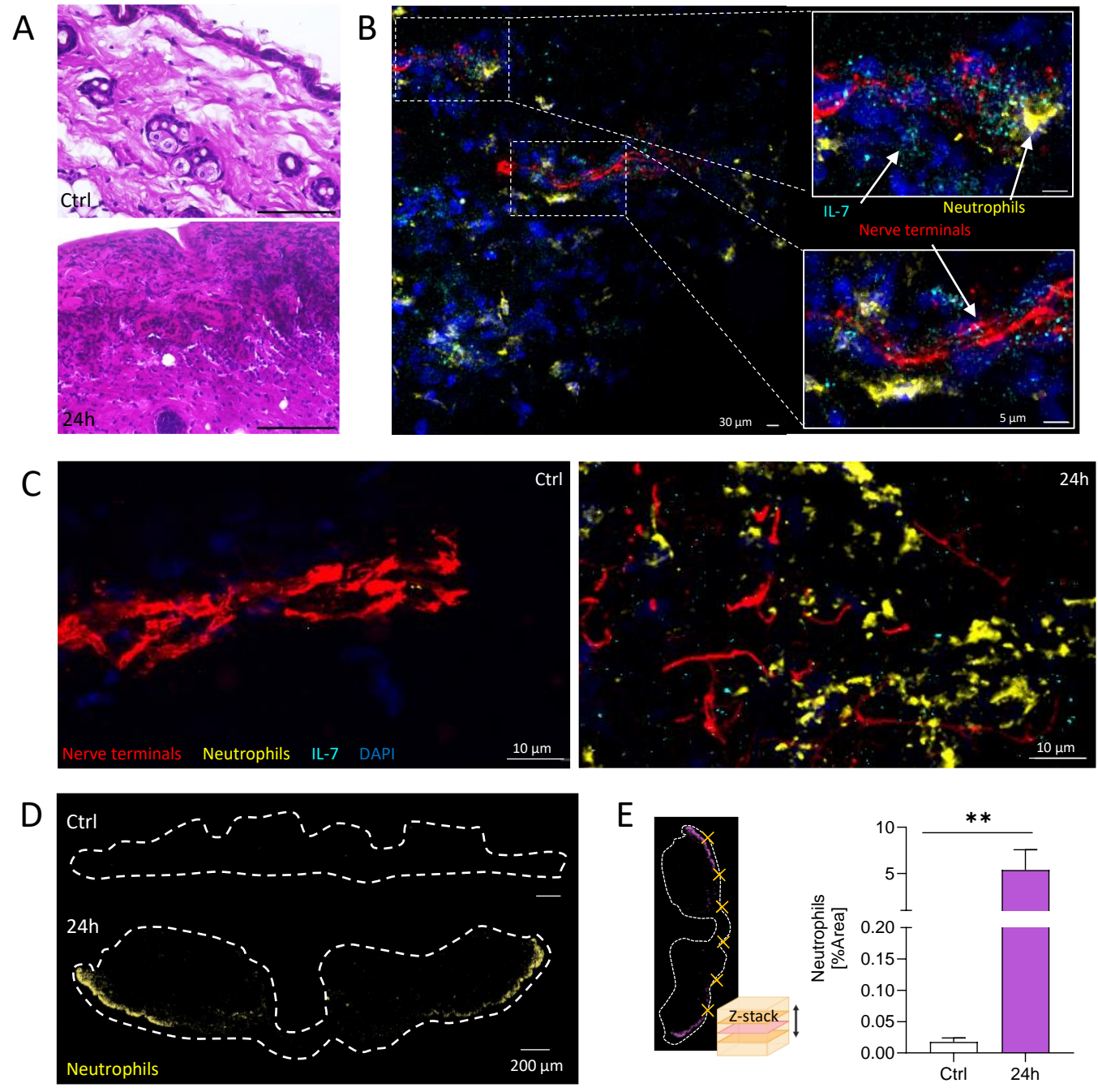

Figure 3

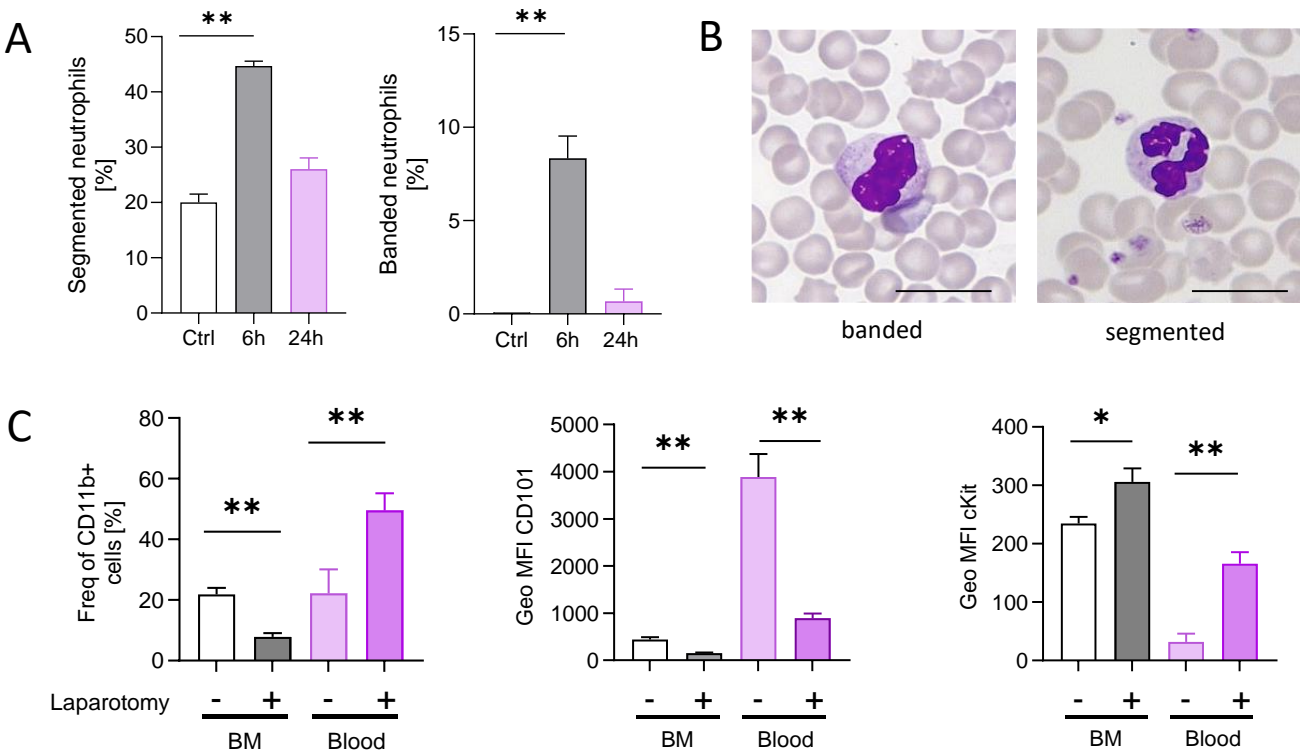

**Figure 4**

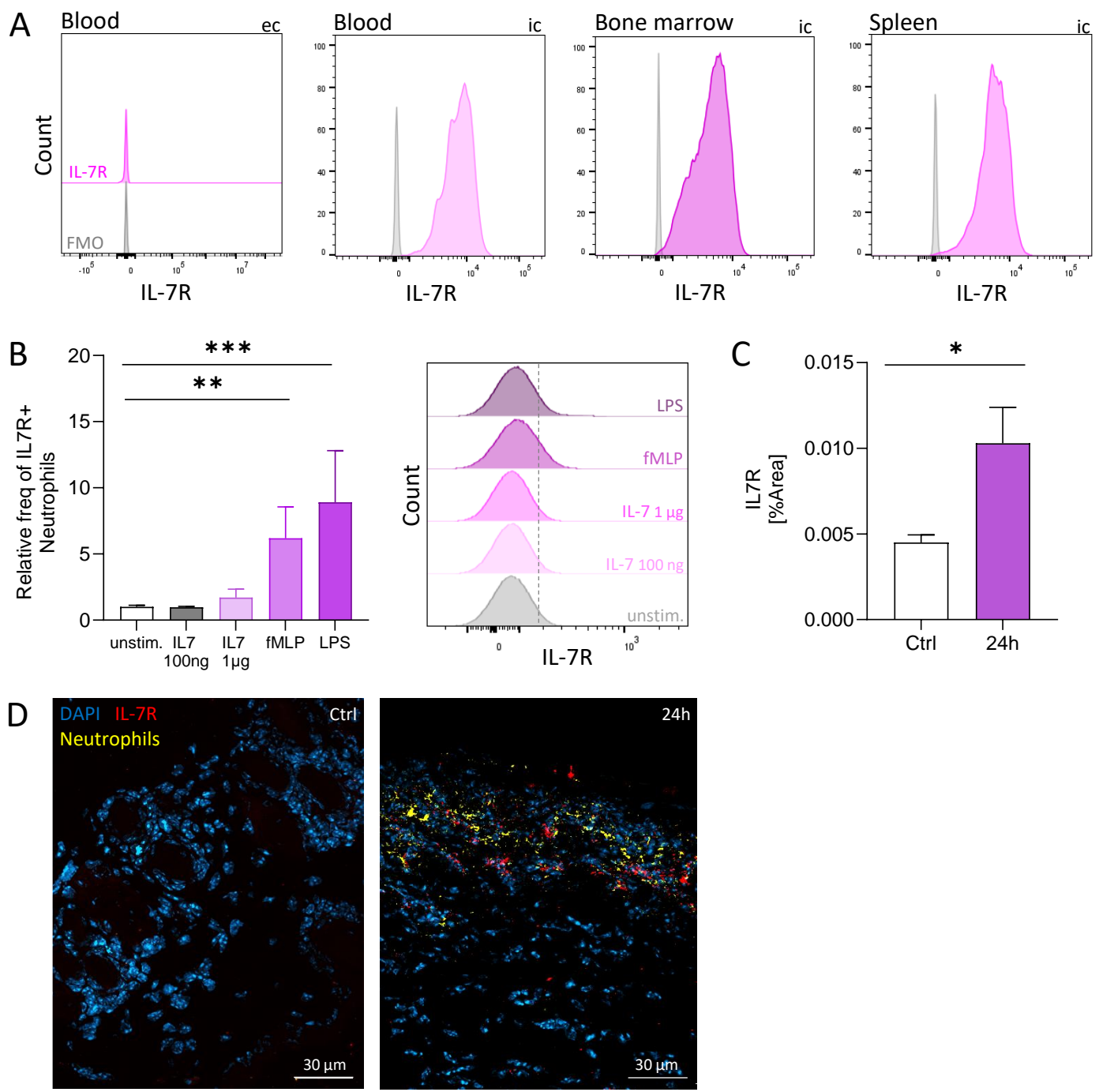

Figure 5

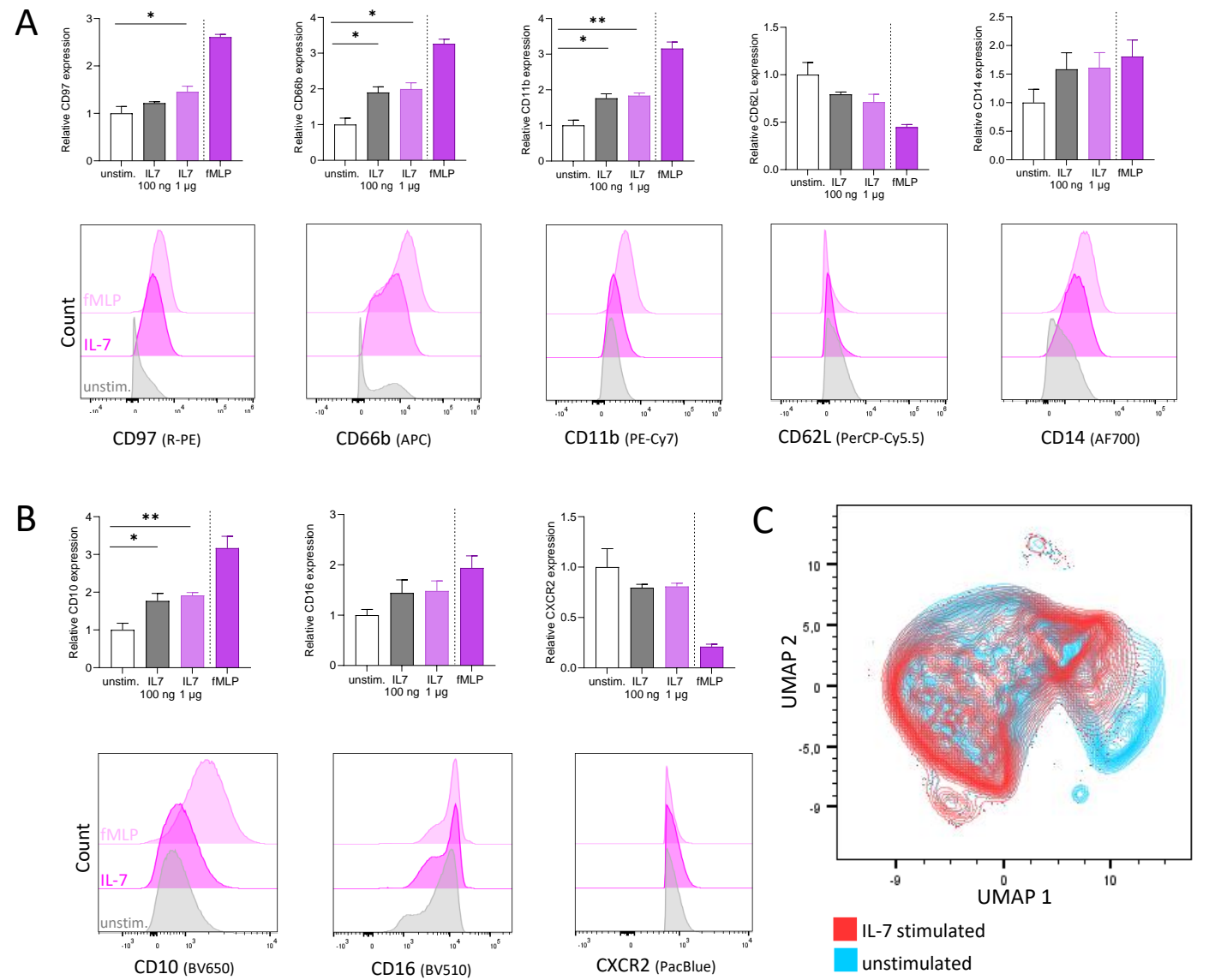

# Supplemental Figure 1

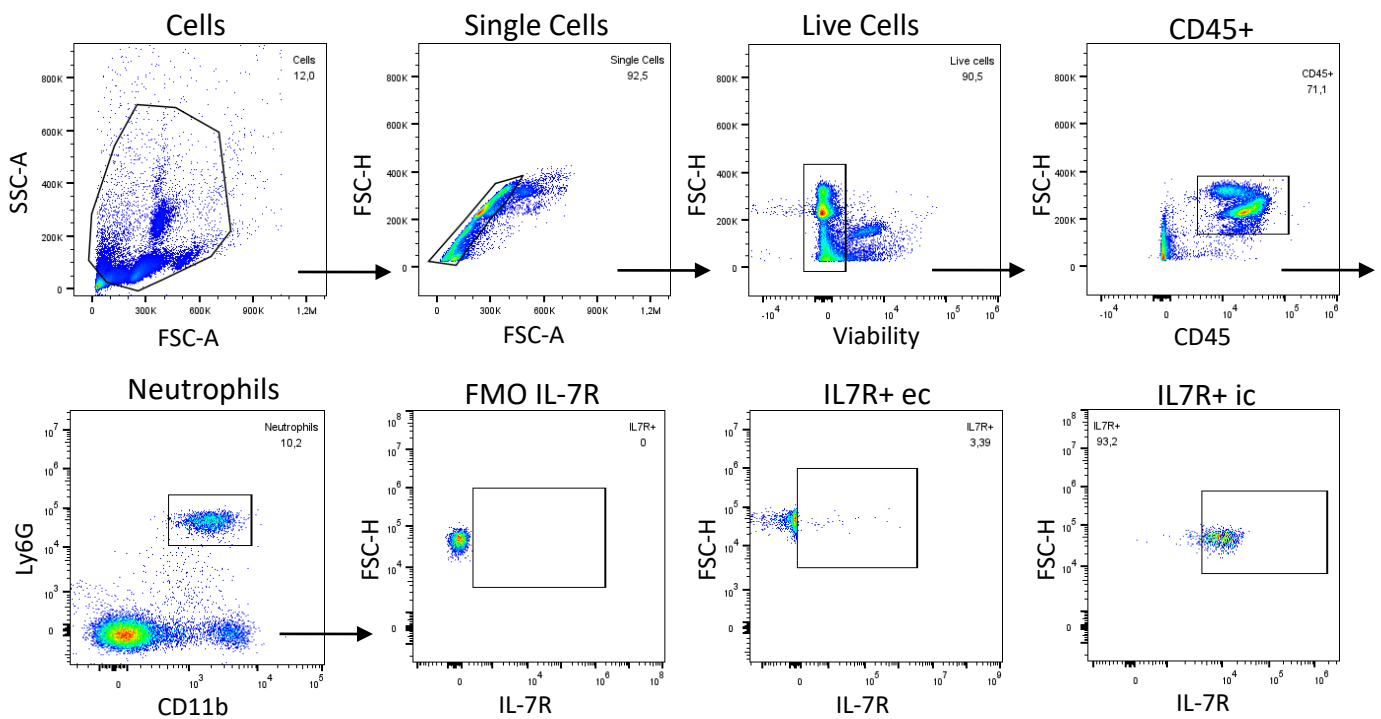

Supplement: Supplementary Data 1 [file mmc1.pdf]
